# Teasing Apart the Effects of Seed Size and Energy Content on Rodent Scatter-Hoarding Behavior

**DOI:** 10.1371/journal.pone.0111389

**Published:** 2014-10-28

**Authors:** Bo Wang, Xiaolan Yang

**Affiliations:** 1 Center for Integrative Conservation, Xishuangbanna Tropical Botanical Garden, Chinese Academy of Sciences, Mengla, Yunnan province, China; 2 Ecotourism Faculty, Southwest Forestry University, Kunming, Yunnan province, China; University of Debrecen, Hungary

## Abstract

Scatter-hoarding rodents are known to play a crucial role in the seed dispersal of many plant species. Numerous studies have indicated that both seed size and the energy content of seeds can affect rodent foraging behavior. However, seed size is usually associated with energy content per seed, making it difficult to isolate how seed size and energy affect rodent foraging preferences. This study used 99 treatments of artificial seeds (11 seed sizes×9 levels of energy content) to tease apart the effect of seed size and energy content on rodent seed-caching behavior. Both seed traits showed significant effects, but their details depended on the stage of the rodent foraging process. Seeds with higher energy content were harvested more rapidly while seed size only had a modest effect on harvest rate. However, after harvesting, seed size showed a much stronger effect on rodent foraging behavior. Rodents’ choice of which seeds to remove and cache, as well as seed dispersal distance, seemed to reflect an optimal seed size. Our findings could be adapted in future studies to gain a better understanding of scatter-hoarding rodent foraging behavior, and the co-evolutionary dynamics between plant seed production and seed dispersers.

## Introduction

Scatter-hoarding rodents are known to play a crucial role in seed dispersal of many plant species, because they usually store large quantities of intact seeds in the soil at many caches [1]–[8]. When scatter-hoarding rodents encounter a seed, they usually face several sequential options: harvest vs. ignore the seed; if they choose to harvest, eat *in* *situ* or move it to elsewhere; upon removal, decide on the removal distance, and whether to eat or cache the removed seed [9]. Basic seed traits, such as seed size [4], [10]–[12], seed geometry [13], [14], chemical content [13], [15]–[18], energy/nutrient content [5], [12], [19], [20], and germination schedule [16], [21], are believed to be essential factors during these important foraging processes.

Seed size is one of the most important seed traits, and its effects on rodent foraging behavior have been extensively discussed. Seed size is usually characterized by two measures – seed mass and seed volume – and both measures are usually positively correlated not only within a species but also among species [22], [23]. Several studies have additionally incorporated seed volume parameters (e.g. area, length, etc.) and their effects on rodent seed foraging. Perea et al. found that animals usually preferred seeds with larger areas than the ones with smaller areas [24], while Holl and Lulow found that neither seed length nor seed weight were correlated with the proportion of seeds predated [22]. However, the majority of the current literature tends to use seed mass. For example, Blate et al. found a significantly negative relationship between seed harvest and seed mass [25], though this was not supported in other studies [15], [26]. Moles et al. found a weakly negative correlation between seed mass and the proportion of seeds harvested at two of their three field sites in Australia, but no significant relationship across 280 species from the global literature [27]. A number of studies have found that heavier seeds were more likely to be removed rather than eaten *in* *situ* [4], [5], [10], [28], [29], while others found medium-massed seeds were more commonly removed than either lighter or heavier seeds [30]. Seed size was also positively correlated to the distance of seed removal by rodents [19], [31], [32].

Why does seed size play such an important role on rodent foraging behavior? First, seed size is usually positively correlated with energy content [4], [12], [19], and rodents usually prefer food of higher energy content [5], [20], [33]. Rodents prefer seeds with higher energy content even when their sizes are the same [12], [20], [33]. Xiao et al. found that the seed energy density – energy per unit weight – plays a key role in rodent foraging decision making process; small *Camellia oleifera* seeds (0.47 g) were harvested as quickly and ultimately the same proportions was removed as large *Lithocarpus harlandii* seeds (3.14 g) [5]. Notably, the smaller *C. oleifera* seeds contained much more fat (51.79% vs. 0.91%) and energy (29.56 vs. 17.11 kJ/g) per unit seed weight than *L. harlandii* [5]. On the other hand, though energy content tends to rise with seed size, larger seeds require more handling time [9], [11], [34]. As such, an optimum seed size may exist that trades off energetic benefits against time/handling costs. For example, rodents preferred to harvest and cache more medium-sized seeds than big and small seeds [9]. Jansen et al. found that for *Carapa procera*, the maximum removal distance for caching was associated with seeds about 29 g, and that dispersal distance decreased with greater seed weight [35].

As discussed above, both seed size and energy content of seed influence scatter-hoarding rodent foraging preferences. However, it is difficult to detect which of the two, seed size or energy content, contributes more, especially across species. Using artificial seeds we can experimentally test the effect of a univariate trait, while keeping other traits constant [9], [12], [20], [33], [36]. For example, Wang et al. used artificial seeds made from clay and peanut powder to investigate foraging preference of scatter-hoarding rodents, and their results showed that seed size affected the probability of a seed being harvested, removed, and cached as well as the seed dispersal distance, while energy content only affected the probability of seed harvest and dispersal distance [9]. However, their experiment only targeted the effects of seed size and energy content separately without their interactions, which might play an important role. Wang and Chen used similar artificial seeds and tried to test the interactive effects of both seed size and energy content on the rodent foraging preference, and their results indicated that seed size is a decisive factor which might attenuate the effects of energy content [12]. However, their experimental designs contained only three seed sizes and two levels of energy content [12], thus a comprehensive understanding of the interactions may not have been obtained.

To further explore how seed size and energy content affect scatter-hoarding rodent foraging preferences, we manipulated seed size and energy content levels by using an artificial seed system [12]. In this study, we created 99 different treatments of artificial seeds with 11 seed sizes, with each seed size having 9 levels of energy content. Our objective was to evaluate the effects of seed size and energy content separately, and also their interactions. Based on the literature and our previous findings, we predict that, (1) rodents will show an increasing preference for higher energy content throughout the entire scatter-hoarding process (i.e. seed harvest, removal, caching and the dispersal distance), as rodents can increase their net rate or efficiency of foraging by choosing higher energy content food; (2) rodents will prefer an optimum seed size (i.e. medium size) during the scatter-hoarding process, as profitability incorporates the tradeoff between handling time and energy content of a seed, both of which scale positively with seed size; (3) compared to energy content, seed size will have a stronger effect on seed removal and dispersal distance, as transporting seeds and subsequently caching them logically require more time and energy than the initial harvest stage, and seed size usually has a direct bearing on handling time and energy cost.

## Materials and Methods

### Ethics Statement

This study was carried out in strict accordance with the Guide for the Care and Use of Laboratory Animals of China. The protocol was approved by the Administrative Panel on the Ethics of Animal Experiments of Xishuangbanna Tropical Botanical Garden, Chinese Academy of Sciences (Permit Number: XTBG2012-006). We signed a contract (No. 20120013) with the Shangri-La Alpine Botanical Garden in 2012 permitting access to the study site for conducting the experiments.

### Study site

The experiment was carried out in October 2012, in a natural forest in the Shangri-La Alpine Botanical Garden, Yunnan province, southwestern China (27°54′ N, 99°38′ E, altitude 3456 m). Here *Pinus densata* is the dominant tree species, and it coexists with several other tree species, e.g. *Pinus armandi*, *Betula delavayi*, *Picea brachytyla*, and *Populus* spp. Sichuan field mouse (*Apodemus latronum*) and Chevrier’s field mouse (*Apodemus chevrieri*) were the two most abundant seed predators/dispersers in the forest; few other animals were found eating or removing the artificial seeds [12], [20], [37]. *Apodemus latronum* was slightly more common than *A. chevrieri* according to our live-trapping census (trap success: 6.7% vs. 1.2%, *B. Wang and X. Yang, unpublished data*). Both rodent species have similar body sizes, about 10 cm in length (without the tail) and only tens of grams in weight. Our previous studies found that both species in the field exhibited similar foraging behavior toward the artificial seeds as they would toward natural seeds in terms of consumption, carrying, and caching [12], [23].

### Study materials-Artificial seeds

We used clay and peanut powder to produce artificial seeds (Fig. S1). Both clay and peanut powder were ground in a mortar until it passed through a 1-mm screen. The resulting powder was mixed and homogenized thoroughly, and then water was added to make a consistent dough. This dough was used to make ball shaped artificial seeds. A 15-cm thin steel thread with a spiral hook on one end was embedded into each seed and connected to a small red plastic tag (2.5 cm in length and 0.7 cm in width) on the the other end. Each tag was numbered for seed identification as well as for detecting where the cached seeds were. After natural drying and hardening, the tags were attached strongly. No tags were found fallen off during the experiments. The length of the steel thread was sufficient to detect the cached seeds since rodents in our study site usually cached the seeds in depths less than 2 cm in the soil, leaving the tags on the surface easy to be detected. For more details see Wang and Chen [12].

### Experimental design

We collected seeds of 11 common plant species in our study forest for analysis to determine the variation in seed size. The mean seed length was 0.8±0.1 cm, with a very narrow range of 0.4–1.4 cm (Table S1). Thus, in order to get a much clearer pattern about rodent foraging preference upon seed size, we used similar experimental design as our previous study [9], in that we expanded the range of artificial seed size (0.2–4.0 cm) beyond what was naturally observed. We devised 99 different treatments of artificial seeds with 11 seed sizes (0.2, 0.4, 0.6, 0.9, 1.2, 1.5, 2.0, 2.5, 3.0, 3.5 and 4.0 cm in diameter) each having 9 levels of energy content (10%, 20%, 30%, 40%, 50%, 60%, 70%, 80% and 90% of peanut powder in weight). The diameter of seeds was used as the measure of seed size in our study. Furthermore, clay is denser than peanut powder so that the percentage of one over the other within a seed would change the overall seed mass but not the volume since the diameter was controlled.

Five plots (2 m×2 m) separated by more than 50 m were established. At each plot, we located 9 seed release points in a 3×3 rectangular grid, with about 1 m between points. Each circle-shaped point was about 15 cm in radius, and the seeds were placed along the circle with the tags pointed outwards. During the experiment, 99 labeled seeds (1 seed for each of the 99 treatments) were randomly placed at each seed release point, resulting in a total of 891 artificial seeds per plot (99 seeds placed in each of the 9 seed release points). Each seed treatment was represented by 45 seeds spread evenly across the 5 plots (9 seeds per plot). In total, 4455 seeds (9 seeds each in 5 plots in each of the 99 seed treatments) were placed in the plots.

### Investigation of seed fate

**Figure 1:**
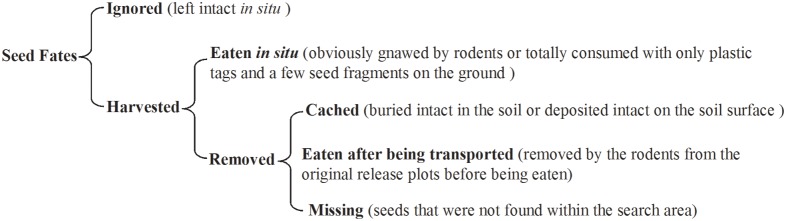
Definitions of different seed fates during the scatter-hoarding process.

To record the number of seeds harvested by rodents we searched for tagged seeds around each plot after days 1, 2, 3, 4, 6, 8, 12, 16, 20, and 28. Based on our previous studies [12], [18], [23], rodents in this forest usually cache seeds less than 20 m from the harvesting site. We therefore, conducted a complete search within 20 m of each plot in all directions to relocate the removed seeds. We also conducted an extra search in a larger area in order to relocate as many of the seeds as possible. We made sure that we employed equal effort for each plot, i.e. two hours of three people for each visit. When we found a cache, we recorded the seed number, seed fate, and its exact location, including the directional angle and distance to the original seed source. We then reburied the seeds in order to minimize disturbance. We also surveyed the seed re-caching process (i.e. cached seeds were often repeatedly excavated and re-cached at another site by rodents). However, we focus only on the first seed movement in this study, as too few seeds were re-cached to conduct any meaningful analysis (only eight secondary caches and one tertiary cache were found). Based on our previous studies [9], seed fates were first divided into two categories (Fig. 1): (1) harvested by rodents and (2) ignored, i.e. left intact *in* *situ*. Harvested seeds were then determined to be either eaten (i.e. obviously gnawed by rodents or totally consumed with only plastic tags and a few seed fragments on the ground) *in* *situ* or removed by rodents; and removed seeds were determined to be cached (buried intact in the soil or deposited intact on the soil surface), eaten after being transported (removed by the rodents from the original release plots before being eaten), or missing (seeds that were not found within the search area).

### Data analysis

We performed several analyses according to our different objectives, using the statistical programming language R (v. 3.1.0) [38]. Generalized linear mixed model (function *glmer*, package ‘lme4’) [39] was used to analyze seed fates with a binomial error distribution and logit link function. The dependent variables were bivariate variables and three models were built representing three stages of the foraging process to analyze whether seeds in the release points were harvested or ignored (Model I), whether the harvested seeds were removed or eaten *in* *situ* (Model II), and whether the removed seeds were cached or eaten by the rodents (Model III). Random effects were considered in a nested structure (seed release point nested in plot). Fixed effects were seed size (including both linear and quadratic terms), energy content and their interactions. We entered the fixed effects as numerical variables and not as factors. We included a quadratic term for seed size (i.e. seed size squared) in the model as seed size presents a tradeoff between energy uptake and handling cost, and thus an optimum seed size might exist. We did not use a quadratic term for energy content as it has no directed relations to the handling cost. Harvest time (i.e. the day on which the seed was found harvested by rodents after being released at the plots) was added as a covariate in Model II and Model III. In Model III we also added distance of seeds removed as a fixed effect. No overdispersion was found by calculating the ratio of residual deviance to the residual degrees of freedom.

A linear Mixed-effects Model (function *lmer*, package ‘lme4’) [39] was used to analyze the effects on seed dispersal distance. We fitted the response variable (distance to seed release point) to a normal distribution after log-transformation. We used the same structure of random effects here as in the models above. Fixed effects were seed size (including both linear and quadratic terms) and energy content, and harvest time was added as covariate. However, we couldn’t get *p*-values from function *lmer*, and we computed the *p*-values by the *lmerTest* package [40] based on Satterthwate’s approximations.

In all cases, to choose the best model and get the appropriate *p* values we did model simplification by using a likelihood ratio test and parsimony criteria. Thus, nonsignificant interactions and terms were removed to achieve the minimal adequate model.

## Results

### Seed harvest

**Figure 2:**
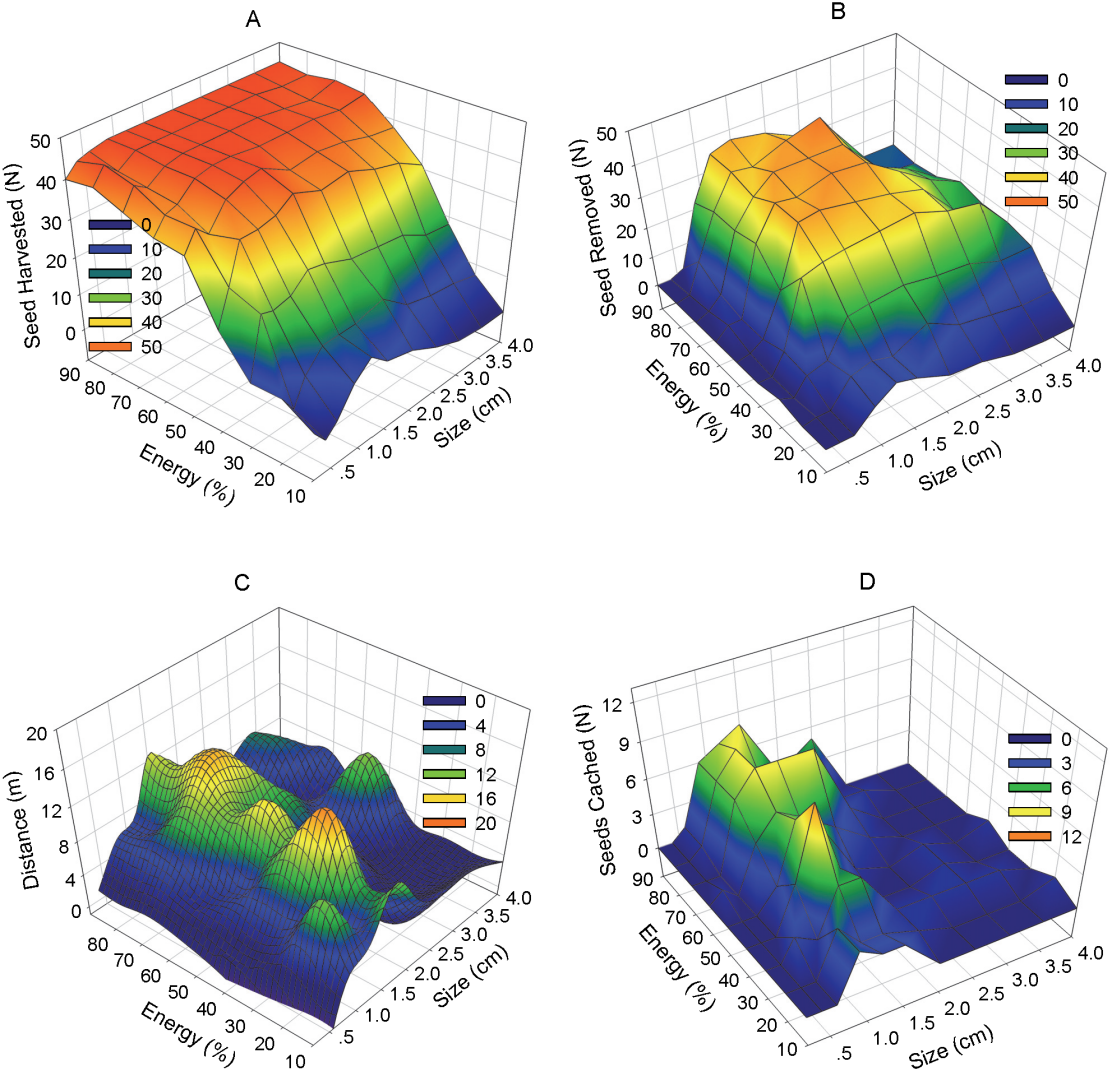
Comparison of fates for seeds with different sizes and energy content levels. There are 99 treatments of artificial seeds with 11 seed sizes×9 levels of energy content. The sample size for each treatment is 45, i.e. 9 seeds×5 plots.

Most of the artificial seeds (71.3%, *n* = 4455) were harvested, while 1279 seeds (28.7%) remained intact at the releasing plots at the end of the experiment. Energy content was a strong predictor of seed harvest (Table 1): seeds with higher energy content were harvested more frequently than seeds with less energy content (Fig. 2A). There was a significant quadratic term in seed size, while the linear term in seed size was not significant (Table 1): the negative sign of the terms meant that the medium-sized seeds were harvested more often than both large- and small-sized seeds (Fig. 2A). There was a significant interaction between the linear term in seed size and energy content (Table 1), and the positive coefficient showed that the slope between the probability of seed harvest and its energy content is larger for large-sized seeds. The negative interactive effect between the quadratic term in seed size and energy content indicated that the quadratic effect of seed size on seed harvest was much stronger for low-energy seeds (Fig. 2A, Fig. S2).

**Table 1:** Summary of the generalized linear mixed models to test the variables affecting seed fate.

Fixed effects	Estimate ± SE	*Z*-value	*P*-value
Harvested vs. Ignored (Model I), *n* = 4455			
Intercept	−4.171±0.883	−4.723	<0.001
Size	0.279±0.443	0.629	0.530
Size Squared	−0.252±0.113	−2.224	0.026
Energy	8.860±0.773	11.465	<0.001
Size×Energy	7.426±1.228	6.050	<0.001
Size Squared×Energy	−1.232±0.311	−3.964	<0.001
Removed vs. Eaten *in* *situ* (Model II), *n* = 3176			
Intercept	−3.586±0.562	−6.383	<0.001
Size	4.542±0.575	7.901	<0.001
Size Squared	−0.834±0.129	−6.458	<0.001
Energy	−0.635±0.778	−0.815	0.415
Day	0.001±0.007	0.110	0.912
Size×Energy	0.841±0.885	0.950	0.342
Size Squared×Energy	−0.398±0.200	−1.990	0.047
Cached vs. Eaten after removed (Model III), *n* = 768			
Intercept	−0.244±1.239	−0.197	0.844
Size	1.781±0.994	1.792	0.073
Energy	−0.938±1.587	−0.591	0.555
Distance	0.300±0.146	2.052	0.040
Size Squared	−0.365±0.176	−2.072	0.038
Day	−0.199±0.025	−7.977	<0.001
Size×Energy	−0.981±0.922	−1.064	0.287
Size×Distance	−0.160±0.097	−1.646	0.100
Energy×Distance	−0.301±0.206	−1.461	0.144
Size×Energy×Distance	0.190±0.134	1.416	0.157

The total number of individuals used (i.e. sample size) in each analysis are shown.

### Seeds removal *vs*. *in* *situ* consumption

Of the 3176 seeds harvested by rodents, 46.3% were eaten *in* *situ*, while 53.7% were removed. Seed removal versus consumption *in* *situ* were highly affected by seed size, as revealed by the linear and quadratic terms in the model. Energy content alone had no significant effect (Table 1). Medium-sized seeds had the highest ratio of removal to *in* *situ* consumption by rodents, followed by large- and small-sized seeds (Fig. 2B). The quadratic term in seed size and energy content also showed a significant interactive effect (Table 1): when seeds were of high energy content, rodents had a strong preference for medium-sized seeds; one the other hand, if seeds were of low energy content, rodents’ preference for medium-sized seeds are not so strong (Fig. 2B).

### Removal distance

Of the 1706 seeds removed from the original releasing plots by rodents, 55% were not found within the search area with their fate unknown. 45% were found and distances from the original releasing plots were measured. Seed size showed significant effects on whether a removed seed was found or not with the result that medium-sized seeds were found least often, followed by large- and small-sized seeds (Table S2). Energy content showed no significant effect (Table S2). However, the absolute quantity of medium-sized seeds we found were still larger than both large- and small-sized seeds (Fig. S3) as much more medium-sized seeds were removed by rodents (Fig. 2B). The mean dispersal distance was 7.5±0.3 m (mean ± SE, 0.3–52.6 m, *n* = 768). Both linear and quadratic terms in seed size significantly affected removal distance, while energy content had no significant effect (Table 2). Medium-sized seeds were dispersed the farthest, followed by large- and small-sized seeds (Fig. 2C). Seed size and energy content showed no interactive effects on seed removal distance. Harvest time showed a significant effect (Table 2); seeds harvested at the beginning of the experiment were removed to a farther distance than seeds harvested later in the experiment.

**Table 2:** Summary of the linear mixed-effects model to test the variables affecting seed dispersal distance (the sample size is *n* = 768).

Fixed effects	Estimate ± SE	*t*-value	*P*-value
Intercept	1.095±0.311	3.518	<0.001
Size	0.954±0.221	4.313	<0.001
Energy	−0.532±0.367	−1.449	0.148
Size Squared	−0.266±0.042	−6.289	<0.001
Day	−0.015±0.006	−2.479	0.013
Size×Energy	0.272±0.170	1.595	0.111

### Seeds cached *vs*. consumption after removal

**Figure 3:**
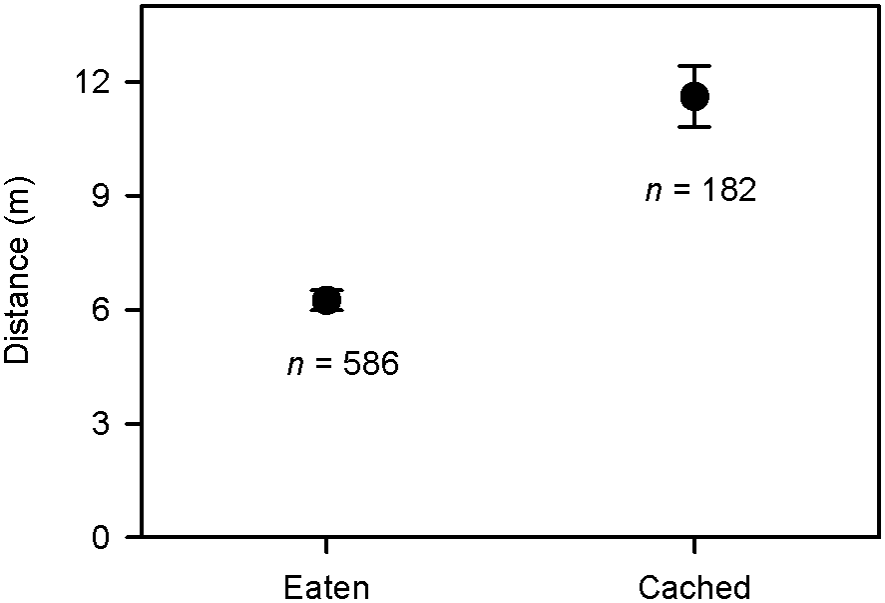
Comparison of dispersal distance between seeds cached and eaten after removal. Numbers below bars are sample sizes.

Of the 768 removed seeds found, most (76.3%) were eaten while only 182 seeds (23.7%) were found cached. Medium-sized seeds were cached rather than being directly consumed much more often than both large- and small-sized seeds (Table 1, Fig. 2D). Energy content showed no significant effect, while dispersal distance was found to be a strong predictor on whether a seed would be cached or eaten after being removed by rodents (Table 1). The farther a seed was dispersed, the more likely it was cached (Fig. 3). Harvest time also showed a highly significant effect (Table 1): seeds removed by rodents at the beginning of the experiment would be cached more often than seeds removed later in the experiment.

## Discussion

In this study, both seed size and energy content were significantly related to rodent foraging behavior, but their effects varied according to the different stages of the scatter-hoarding process. In general, rodents preferred medium-sized and high energy content seeds. High energy seeds were harvested at much more rapid rates while seed size had a limited effect on harvest rate. On the other hand, seed size was more important for the remaining scatter-hoarding stages: seed removal or consumption *in* *situ*, decision on dispersal distance, and seed caching or consumption after removal.

As discussed in the introduction, seed size is usually estimated by seed mass and seed volume. In this study, we used seed volume (i.e. seed diameter) to indicate the size of our artificial seed as seed diameter is much more easy to artificially control, though most of the current studies used seed mass [4], [5], [19], [29], [31]. However, rodent foraging behavior responds similarly to seed mass and seed volume [22], [23]. For example, Holl and Lulow found that neither seed length nor seed weight was correlated with the proportion of seed predation [22], while Wang et al. found both seed mass and seed length were positively correlated to the probability of seeds being harvested and cached [23]. Furthermore, seed mass and seed volume are usually positively related to each other and can both be measures of the energy content of a seed [10], [12], [19], [23], [24]. So, in the following discussion, we just used the word ‘seed size’.

Numerous studies have shown significantly positive relationships between seed size and scatter-hoarding rodent preferences for seed harvest, removal, caching and dispersal distance [4], [5], [19], [29], [31]. Our results indicated the existence of an optimal seed size, specifically medium-sized seeds, which maximized the energetic benefit to rodents while reducing handling costs. Similar results were also indicated by several other studies [9], [30], [35]. Optimal foraging models indicate animals maximize the net rate [34] or efficiency of foraging [41] with time as the primary limiting factor. Although larger seeds might contain more energy, manipulating larger seeds usually requires longer handling time [42]. Thus choosing the largest seed may not maximize the net rate of energy gain. Furthermore, increased handling time may also expose animals to greater predation risk [43]. The tradeoff between foraging benefit and predation risk may also prevent rodents from choosing the largest seeds. Perea et al. also indicated that higher predation risk might decrease the seed handling time by wood mice, thus influencing their seed selection preference [44]. In this study, we have a much larger range of seed size in the artificial seed system than the natural species in the field. It may be argued that our finding that rodent prefer medium-sized seeds is an artifact which comes from that medium-sized artificial seeds are the maximum size a rodent in our study area can meet. Nevertheless, we should note that the observed preference for medium-sized seeds must be seen in relation to rodent body size, as the rodents we surveyed were quite small (only about 10 cm in length without the tail and tens of grams in weight). Rodent body size would mediate whether or not there is a seed size beyond which handling and transport become increasingly difficult and expensive [9], [11]. However, the rodents in this study were indeed able to handle these large seeds although they were much larger than the maximum sized natural seeds. The appendix of the artificial seeds (*i.e*. the steel thread and plastic tag) might make the handling and transporting of this large seeds easier for the small rodents. Furthermore, we reanalyzed our data by only consider artificial seeds falling within the natural seed size limits (0.4–1.5 cm), and the results also showed a negative sign of quadratic term in seed size for seed harvest, removal and caching (Table S3), but with a weaker pattern than that with the whole range (Fig. S4); there was no significant negative sign of quadratic term in seed size for the dispersal distance (Table S4, Fig. S4).

Many studies have indicated that the energy content of seeds could influence seed harvest, seed removal, and also the dispersal distance [5], [19], [23], [28], [45]. Our results suggest that higher energy content rapidly increased seed harvest rate, but the extremely low energy content of our most energy poor artificial seeds may have been the driving force behind much of this observation. Energy content showed no significant effect on seed removal, seed caching and the dispersal distance, and this could be the consequence of rodents harvesting mainly the high energy seeds, hence, leaving no variation in seed energy content for the later stages of the process. However, there might be an alternative explanation, i.e. the overwhelming effects of seed size may attenuate the effect of energy content [12]. Seed size usually has a direct bearing on handling time, and thus foraging efficiency [34,41,42], especially for the small-bodied mouse species in our study. Kerley and Erasmus also found that rodents avoided high energy seeds which tended to be large and with long handling times [46].

Our results showed that dispersal distance influenced whether a seed would be cached or eaten after removal, with the result that seeds dispersed farther were more likely to be cached rather than eaten. Transporting seeds has a high energy cost, which may positively relate to dispersal distance, which may be particularly true for small-bodied rodents. It would be reasonable to assume that rodents should cache a seed after long distance transporting because of the energy investment already incurred for carrying the seed. However, this is not the case during the re-caching process because seed re-caching increases their dispersal distance but decreases the survival probability of the cached seed [7], [47]. This might be because re-caching increases the number of seed-rodent encounters, thus increasing the probability of seed consumption [7], [47]. While seed size, energy content and dispersal distance are important for caching, other potential factors may also play an important role on the foraging decision of caching vs. eating the seed, e.g., seed tannin content, destination microhabitat, seed germination schedule, etc. [7], [16], [21], [48]. Furthermore, our study suggested that seeds harvested at the beginning of the experiment would be transported to a farther distance than seeds harvested later in the experiment, with a higher probability of being cached rather than eaten. This significantly negative effect of harvest time on dispersal distance and caching probability might be indirectly affected by seed traits. Rodents usually harvested their preferred seeds (e.g. seeds with more fat or energy) more rapidly [5], [20], [35], and this were also true in our case. Rodents harvested seeds with medium sizes or more energy content more rapidly (Fig. S5).

In our experiment, more than half the removed seeds (55.0%, *n* = 1706) were not found, thus, their fate is unknown. Seed size showed significant effects on whether a removed seed was found or not with the result that medium-sized seeds were found least often, followed by large- and small-sized seeds (Table S2). Energy content showed no significant effect (Table S2). This difference in seed recovery rates would, if anything, lead to an underestimation of the proportion of seeds that were cached and the dispersal distance, particularly so for medium-sized seeds. Some studies indicated that these missing seeds might be dispersed beyond the search radius [4], [49], [50], which may be true in our case too. However, it is also possible that not all missing seeds were cached beyond the search radius. In our study area, seeds were usually found transported by rodents with less than 20 m, even when there was a much smaller proportion (e.g. 9.6% and 18.3%) of missing seeds [23], [37]. The dominant rodents in our study site are usually small bodied, which might be a limiting factor for transporting seeds to far distances. Larder-hoarding behavior might be an alternative explanation for the missing seeds [51], [52]. In our study, the line tied to the tags was about 15 cm, thus the tags might not be detected on the surface if rodents carried the seeds to deep burrows. This later presumption might be supported by the fact that medium-sized seeds were most frequently missed which is the preferred seed size by rodents.

Both seed size and energy content played important roles on the scatter-hoarding rodent foraging process, but varied according to the different stages. By using artificial seeds, we effectively teased apart the effect of seed size and energy content on the initial seed handling (i.e. seed harvest stage). In the later stages of the rodent foraging process, seed size played a critical role while energy content was of minimal importance. However, only relatively few low energy seeds were left after the seed harvest stage which may have biased the effects of energy in the later stages. Nevertheless, our findings shed light on scatter-hoarding rodent foraging behavior, and more specifically, demonstrate that there maybe important evolutionary tradeoffs imposed on seed size of rodent-dispersed species.

## Supporting Information

Figure S1 **Artificial seeds made from clay and peanut powder.**
(JPG)

Figure S2 **Relations between seed size and seed harvest at different levels of energy content.**
(JPG)

Figure S3 **Number of seeds being found after removal with different sizes and energy content levels.**
(JPG)

Figure S4 **Comparison of seed fates with different sizes and energy content levels for the seeds within the natural seed size limits.**
(JPG)

Figure S5 **Relations between harvest time and seed size and energy content, respectively.**
(JPG)

Table S1 **Seed length of 11 common species in the study site.**
(DOC)

Table S2 **Summary of the generalized linear mixed models to test the variables affecting whether a removed seed was found or not.**
(DOC)

Table S3 **Summary of the generalized linear mixed models to test the variables affecting the fates of the seeds within the natural seed size limits.**
(DOC)

Table S4 **Summary of the linear mixed-effects model to test the variables affecting the dispersal distance of the seeds within the natural seed size limits.**
(DOC)
