# Supplementary figures and images for: Teasing Apart the Effects of Seed Size and Energy Content on Rodent Scatter-Hoarding Behavior
Source: PLoS One. 2014 Oct 28;9(10):e111389. doi: 10.1371/journal.pone.0111389 (PMC4211888; doi:10.1371/journal.pone.0111389)

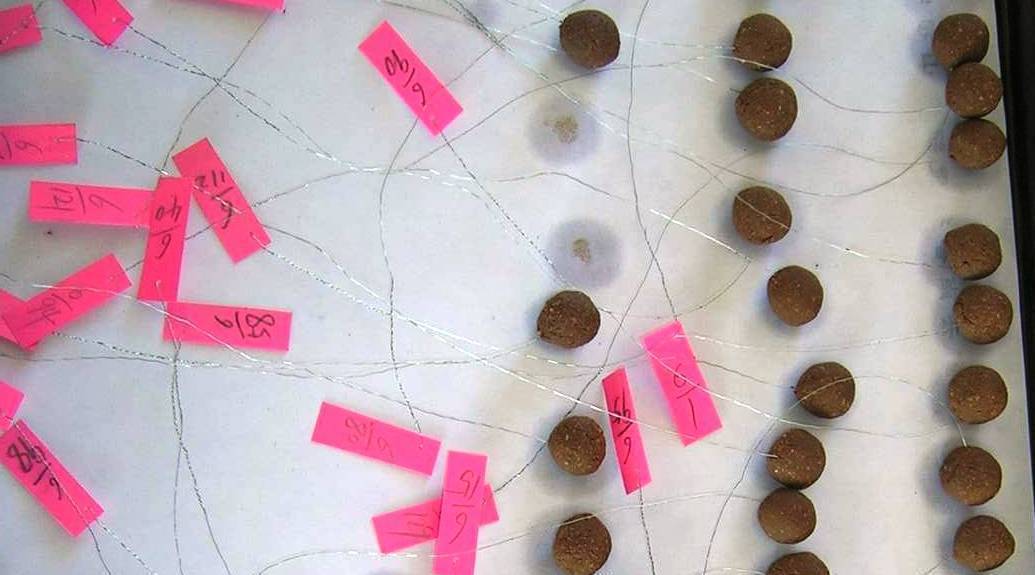

Supplement: Figure S1 — Artificial seeds made from clay and peanut powder. (JPG) [file pone.0111389.s001.jpg]

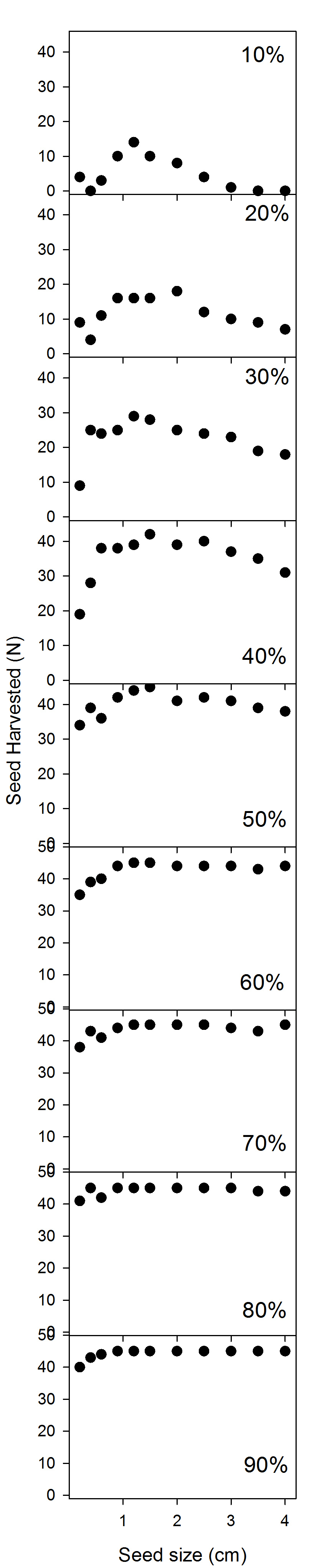

Supplement: Figure S2 — Relations between seed size and seed harvest at different levels of energy content. (JPG) [file pone.0111389.s002.jpg]

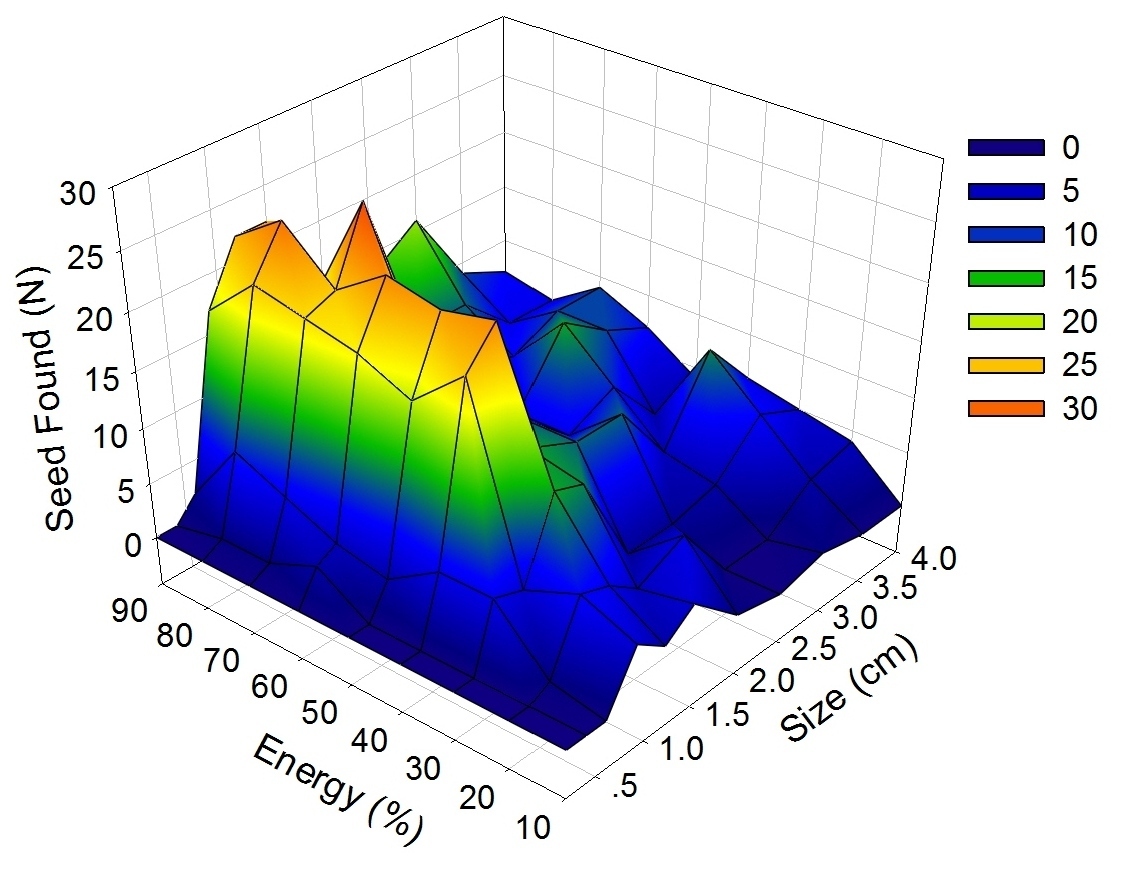

Supplement: Figure S3 — Number of seeds being found after removal with different sizes and energy content levels. (JPG) [file pone.0111389.s003.jpg]

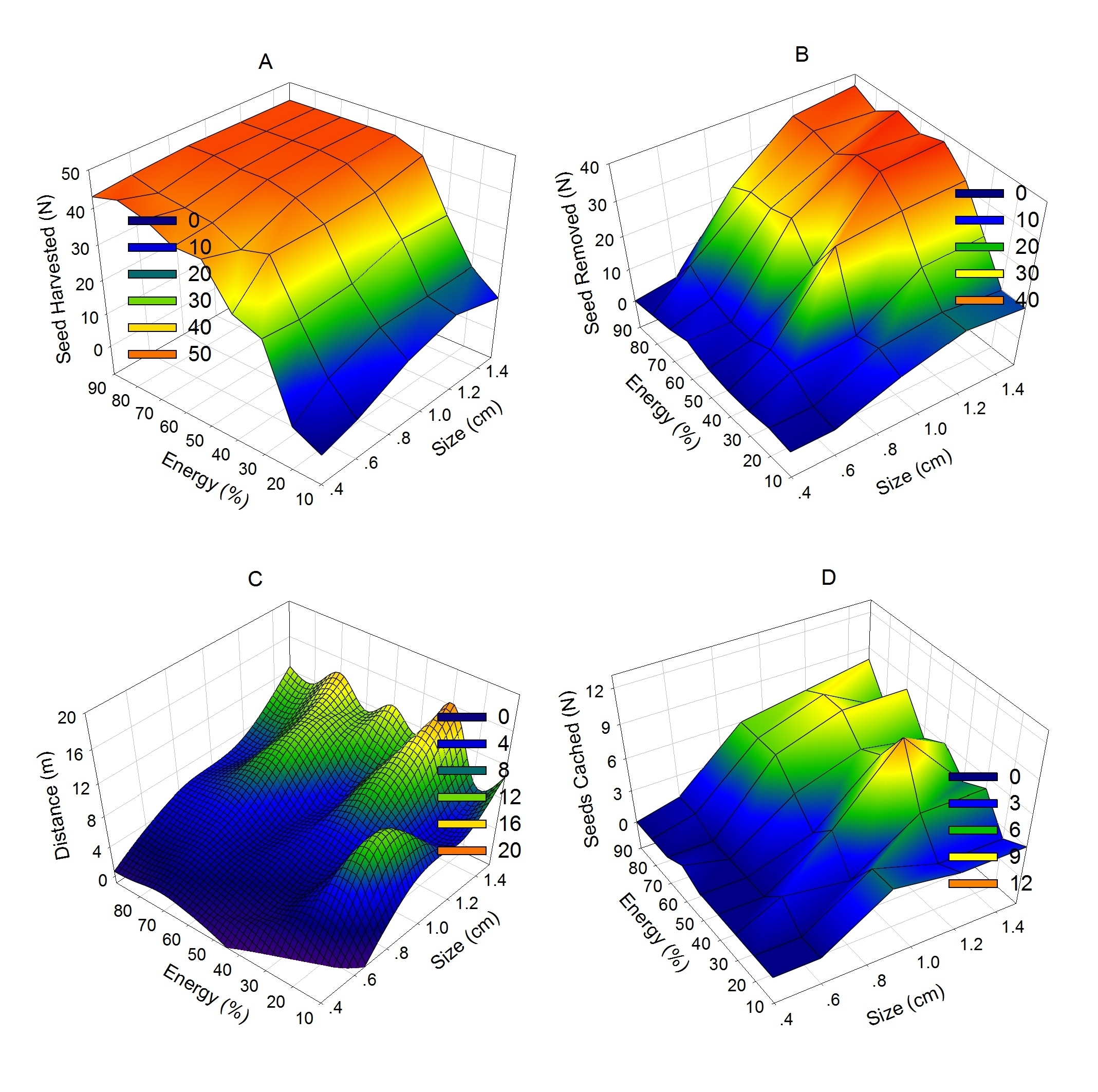

Supplement: Figure S4 — Comparison of seed fates with different sizes and energy content levels for the seeds within the natural seed size limits. (JPG) [file pone.0111389.s004.jpg]

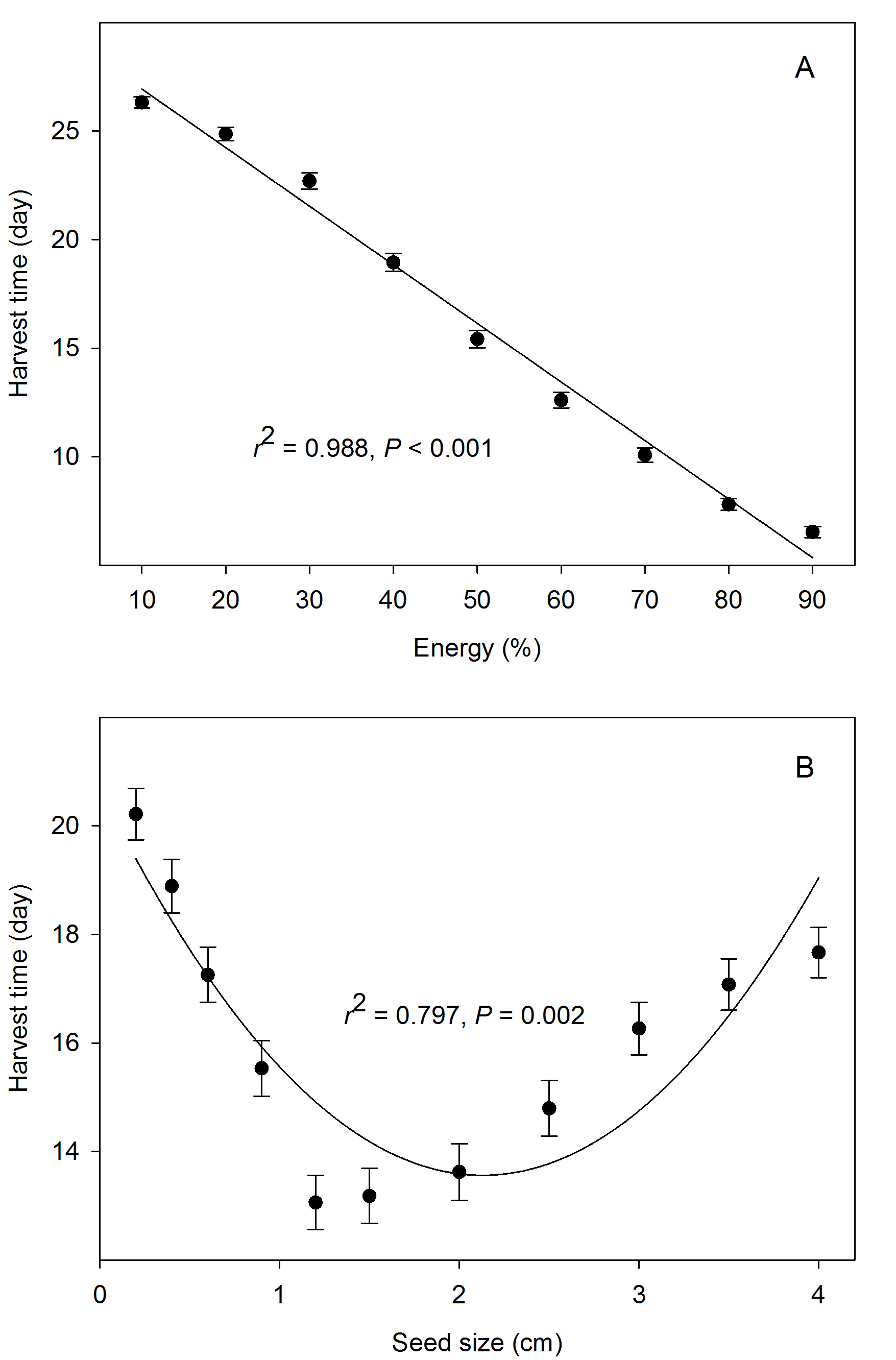

Supplement: Figure S5 — Relations between harvest time and seed size and energy content, respectively. (JPG) [file pone.0111389.s005.jpg]
