# Supplementary material for: Teasing Apart the Effects of Seed Size and Energy Content on Rodent Scatter-Hoarding Behavior
Source: PLoS One. 2014 Oct 28;9(10):e111389. doi: 10.1371/journal.pone.0111389 (PMC4211888; doi:10.1371/journal.pone.0111389)
Supplement: Table S1 — Seed length of 11 common species in the study site. (DOC) [file pone.0111389.s006.doc]

**Table S1 Seed length (Mean ± SD) of 11 common species in the study site.**

| Family | Species | Length (cm)  *n* = 30 |
| --- | --- | --- |
| Cucurbitaceae | *Hemsleya pedunculosum* | 1.13 ± 0.07 |
| Dipsacaceae | *Dipsacus chinensis* | 0.49 ± 0.02 |
| Iridaceae | *Iris bulleyana* | 0.46 ± 0.06 |
| Pinaceae | *Abies forrestii* | 1.13 ± 0.15 |
| Pinaceae | *Pinus armandii* | 1.36 ± 0.07 |
| Pinaceae | *Pinus densata* | 0.49 ± 0.06 |
| Podophyllaceae | *Sinopodophylum hexandrum* | 0.44 ± 0.05 |
| Ranunculaceae | *Anemone sp.* | 0.89 ± 0.05 |
| Ranunculaceae | *Thalictrum uncatum* | 0.61 ± 0.05 |
| Rosaceae | *Cotoneaster sp.* | 0.56 ± 0.03 |
| Rosaceae | *Rosa omeiensis* | 0.71 ± 0.06 |
