# Supplementary material for: Teasing Apart the Effects of Seed Size and Energy Content on Rodent Scatter-Hoarding Behavior
Source: PLoS One. 2014 Oct 28;9(10):e111389. doi: 10.1371/journal.pone.0111389 (PMC4211888; doi:10.1371/journal.pone.0111389)
Supplement: Table S2 — Summary of the generalized linear mixed models to test the variables affecting whether a removed seed was found or not. (DOC) [file pone.0111389.s007.doc]

**Table S2 Summary of the** **generalized linear mixed models to test the variables affecting whether a removed seed was found or not (the sample size is *n* = 1706).**

| Fixed effects | Estimate ± SE | *Z*-value | *P*-value |
| --- | --- | --- | --- |
| Intercept | -3.715 ± 0.512 | -7.262 | <0.001 |
| Size | 3.797 ± 0.344 | 11.026 | <0.001 |
| Energy | -0.559 ± 0.606 | -0.922 | 0.357 |
| Size Squared | -0.672 ± 0.064 | -10.530 | <0.001 |
| Size×Energy | -0.188 ± 0.270 | -0.691 | 0.490 |
