# Supplementary material for: Teasing Apart the Effects of Seed Size and Energy Content on Rodent Scatter-Hoarding Behavior
Source: PLoS One. 2014 Oct 28;9(10):e111389. doi: 10.1371/journal.pone.0111389 (PMC4211888; doi:10.1371/journal.pone.0111389)
Supplement: Table S3 — Summary of the generalized linear mixed models to test the variables affecting the fates of the seeds within the natural seed size limits. (DOC) [file pone.0111389.s008.doc]

**Table S3 Summary of the generalized linear mixed models to test the variables affecting the fates of the seeds within the natural seed size limits (0.4-1.5 cm).** The total number of individuals used (*i.e*. sample size) in each analysis are shown.

| Fixed effects | Estimate ± SE | *Z*-value | *P*-value |
| --- | --- | --- | --- |
| Harvested vs. Ignored (Model I), *n* = 2025 | | | |
| Intercept | -7.952 ± 1.464 | -5.431 | <0.001 |
| Size | 9.584 ± 2.963 | 3.235 | 0.001 |
| Size Squared | -4.998 ± 1.576 | -3.172 | 0.002 |
| Energy | 18.205 ± 3.411 | 5.337 | <0.001 |
| Size×Energy | -16.255 ± 8.538 | -1.904 | 0.057 |
| Size Squared×Energy | 11.991 ± 4.764 | 2.517 | 0.012 |
| Removed vs. Eaten *in situ* (Model II), *n* = 1497 | | | |
| Intercept | -6.410 ± 0.988 | -6.486 | <0.001 |
| Size | 13.182 ± 1.498 | 8.799 | <0.001 |
| Size Squared | -5.017 ± 0.654 | -7.674 | <0.001 |
| Energy | -2.313 ± 1.102 | -2.099 | 0.036 |
| Day | -0.043 ± 0.011 | -3.828 | <0.001 |
| Size×Energy | 1.289 ± 0.968 | 1.331 | 0.183 |
| Cached vs. Eaten after removed (Model III), *n* = 466 | | | |
| Intercept | -4.219 ± 2.763 | -1.527 | 0.127 |
| Size | 9.129 ± 3.852 | 2.370 | 0.018 |
| Energy | 0.638 ± 3.579 | 0.178 | 0.859 |
| Distance | 0.278 ± 0.261 | 1.063 | 0.288 |
| Size Squared | -3.443 ± 1.623 | -2.122 | 0.034 |
| Day | -0.183 ± 0.028 | -6.512 | <0.001 |
| Size×Energy | -2.605 ± 3.007 | -0.866 | 0.386 |
| Size×Distance | -0.156 ± 0.195 | -0.797 | 0.426 |
| Energy×Distance | -0.433 ± 0.382 | -1.135 | 0.257 |
| Size×Energy×Distance | 0.309 ± 0.287 | 1.076 | 0.282 |
