# Supplementary material for: Teasing Apart the Effects of Seed Size and Energy Content on Rodent Scatter-Hoarding Behavior
Source: PLoS One. 2014 Oct 28;9(10):e111389. doi: 10.1371/journal.pone.0111389 (PMC4211888; doi:10.1371/journal.pone.0111389)
Supplement: Table S4 — Summary of the linear mixed-effects model to test the variables affecting the dispersal distance of the seeds within the natural seed size limits. (DOC) [file pone.0111389.s009.doc]

**Table S4 Summary of the linear mixed-effects model to test the variables affecting the dispersal distance of the seeds within the natural seed size limits (0.4-1.5 cm).** The total number of individuals used (*i.e*. sample size) is *n* = 466.

| Fixed effects | Estimate ± SE | *t*-value | *P*-value |
| --- | --- | --- | --- |
| Intercept | 0.918 ± 0.609 | 1.509 | 0.132 |
| Size | 1.027 ± 0.481 | 2.137 | 0.033 |
| Energy | -0.798 ± 0.918 | -0.870 | 0.385 |
| Day | -0.025 ± 0.007 | -3.411 | <0.001 |
| Size×Energy | 0.333 ± 0.756 | 0.440 | 0.660 |
